# Combining attention training with cognitive-behavior therapy in Internet-based self-help for social anxiety: study protocol for a randomized controlled trial

**DOI:** 10.1186/1745-6215-14-68

**Published:** 2013-03-08

**Authors:** Johanna Boettcher, Gerhard Andersson, Per Carlbring

**Affiliations:** 1 Department of Clinical Psychology and Psychotherapy, Freie Universität Berlin, Berlin, Germany; 2 Department of Psychology, Stockholm University, Stockholm, Sweden; 3 Department of Behavioural Sciences and Learning, Linköping University, Linköping, Sweden; 4 Department of Clinical Neuroscience, Karolinska Institutet, Stockholm, Sweden

**Keywords:** Cognitive bias modification, Internet-administered cognitive-behavior therapy, Social phobia, Social anxiety disorder

## Abstract

**Background:**

Guided Internet-based cognitive-behavioral therapy (ICBT) has been found to be effective for social anxiety disorder (SAD) by several independent research groups. However, since the extent of clinically significant change demonstrated leaves room for improvement, new treatments should be developed and investigated. A novel treatment, which has generally been found to be effective, is cognitive bias modification (CBM). This study aims to evaluate the combination of CBM and ICBT. It is intended that two groups will be compared; one group randomized to receiving ICBT and CBM towards threat cues and one group receiving ICBT and control training. We hypothesize that the group receiving ICBT plus CBM will show superior treatment outcomes.

**Methods/design:**

Participants with SAD (*N* = 128), will be recruited from the general population. A composite score combining the scores obtained from three social anxiety questionnaires will serve as the primary outcome measure. Secondary measures include self-reported depression and quality of life. All treatments and assessments will be conducted via the Internet and measurement points will be baseline, Week 2, post-treatment, and 4 months post-treatment.

**Discussion:**

There is no direct evidence of the effects of combining CBM and ICBT in SAD. Adding attention-training sessions to ICBT protocols could increase the proportion of participants who improve and recover through Internet-based self-help.

**Trial registration:**

ClinicalTrials.gov:NCT01570400

## Background

Social anxiety disorder (SAD) is one of the most common mental disorders in Western civilizations. Estimations of lifetime prevalence rates vary between 6.6% in Europe [1] and 12.1% in the USA [2]. The disorder is characterized by the fear and avoidance of being judged or criticized by others. It often takes a chronic course (for example, [3,4]) and is associated with major impairments in quality of life and daily functioning. Individuals with SAD are less likely to be married than healthy controls, and are more likely to be unemployed or hold jobs beneath their qualification [1]. Cognitive-behavior therapy (CBT) has proven effective in addressing the symptoms of this chronic and disabling disorder (for an overview, see [5]). However, the treatment rate is low with only about one-quarter to one-third of people with SAD receiving treatment [6,7]. This low treatment rate is certainly partly due to restricted healthcare facilities. On the other hand, the disorder-specific fear of social situations offers a further explanation of why individuals with SAD take up to 20 years to consult a professional [8]. Olfson *et al*. [9] found that about 20% of those individuals with SAD who do not seek treatment avoid this because of their fear of what others might think of them.

Internet-based treatments offer a solution to these barriers to seeking treatment [10]. Internet-based self-help combines the advantages of high availability and easy accessibility. Furthermore, the feared face-to-face confrontation with a clinician can be circumvented, as all assessments and interventions are conducted via Internet and telephone. Internet-based cognitive-behavioral self-help has proven effective in reducing symptoms of social anxiety in several randomized controlled trials (for example, [11-15]). The results are maintained up to 5 years after completion of the treatment [16,17]. Andrews, Davis, and Titov [18] and Hedman and coworkers [19] could also show that ICBT was as effective as face-to-face therapy when studied in the clinical routine. Most randomized controlled trials of ICBT in SAD show encouragingly large effect sizes. These, however, do not capture the extent of clinically significant change. Therefore, many studies also report the proportions of improved and recovered participants. These proportions mostly range from 50% to 65% (for example, [11,12,20]). It seems that the majority of participants benefit from the Internet-based treatment. At the same time, a substantial number of participants do not respond to the self-help manual. The applied CBT rationales are based on the cognitive model of Clark and Wells [21]. They address safety behaviors, avoidance, negative thoughts, and self-focused attention. However, cognitive models also emphasize the role of biases in information processing not addressed in CBT manuals. Biases in interpretation and attention processes are thought to be crucial in the maintenance of SAD. The investigation of biases in the allocation of attention, in particular, has been the focus of many experimental studies. These suggest that socially anxious individuals differ from nonanxious controls in attention to social threat information. Most of these studies applied either the emotional Stroop or the dot-probe paradigm [22].

Consistently, findings of the emotional Stroop test showed higher response latencies for social threat words compared with neutral words, suggesting an impaired ability to disengage attention from threat in SAD [23-26]. Results of the dot-probe paradigm are more mixed. Studies differ in the type of stimulus (words vs. faces) and the length of stimulus presentation (80 ms, 200 ms, 500 ms, >1000 ms). In this paradigm, two stimuli (for example, one neutral and one social threat word) are simultaneously displayed on a computer screen followed by a probe in the location of one of the stimuli. Faster responses to probes in the location of the social threat word compared with responses to neutral words indicate a biased attention towards threat. Faster responses to the neutral cue indicate a biased attention away from threat (avoidance of threat cues). Taken together, results of dot-probe studies suggest that there is some evidence of an attention bias towards threat early in cue detection (≤500 ms) but no attention bias at longer presentation times [27-30]. Fewer studies suggest that there may be an attention bias away from threat [31,32]. This is supported by an eye-tracking study revealing initial and sustained attentional avoidance of social threat faces [33].

The maintaining role of biased attention processes has further been demonstrated in studies manipulating this bias. Most studies that considered modification of attention bias concentrated on facilitating disengagement from threat. Amir *et al*. [34] and Schmidt *et al*. [35] both evaluated an attention-training program that directed attention away from threat for individuals with SAD. The training program was based on the dot-probe paradigm and included eight 20-minutes sessions. Using self-report and clinician-rated measures of social anxiety, both Schmidt *et al.*[35] and Amir *et al.*[34] reported very positive results for the attention-training group in comparison with a control group. In a recent trial, Heeren *et al*. [36] demonstrated that an attention-training program directed towards nonthreatening cues was effective in reducing self-report, behavioral, and physiological measures of social anxiety. Similar studies in nonclinical samples of socially anxious individuals supported these positive results (for example, [37-39]). Klumpp and Amir [38] compared two different attention-training programs. One program trained participants to focus less on social threat cues (reducing the bias towards threat), while the other program trained individuals to focus more on threat cues (reducing the bias away from threat). In trials on an Internet-based attention-training program, Boettcher, Berger, and Renneberg [40], as well as Carlbring and colleagues [41], could not find significant effects when applying an attention-training program that aimed at reducing attention bias towards threat. However, in a subsequent trial, Boettcher and colleagues [42] reported good effects for a modified, Internet-based attention-training program. The authors compared several attention-training programs. Patients with SAD benefited most from a 14-day, web-based program that involved attention training towards negative cues. This modified-attention program targeted the attentional *avoidance* of threat cues in socially anxious individuals.

First studies on the predictive value of attention bias in cognitive-behavior therapy show that individuals with a pronounced attention bias benefit less from CBT for SAD. Legerstee *et al*. [43] and Price *et al*. [44] both showed that greater attention-bias scores prior to the intervention were associated with higher post-treatment anxiety scores in CBT. Pishyar *et al*. [45] and Lundh and Öst [46] found that CBT interventions reduced biases in attention processes in individuals with SAD and that this reduction of the attention bias was associated with changes in social anxiety. These findings imply that a combination of attention training and CBT could be beneficial in reducing symptoms of SAD. Even if CBT manuals do not focus explicitly on the attention bias, patients are encouraged to process all social cues (negative, positive, and neutral) in exposure exercises and behavioral experiments. As Rodebaugh *et al*. [5] concluded, all therapies for SAD share the assumption that change will occur through the experience of a social situation as it, “is actually like, as opposed to how clients fear or think it will be.”

Until now, there has been no direct evidence of the advantages of combining attention training with CBT in SAD. However, Amir and Taylor [47] showed promising results in a small study targeting generalized anxiety disorder. Adding attention-training sessions to ICBT protocols could increase the proportion of participants who improve and recover through Internet-based self-help.

This study aims at evaluating the combination of attention training and Internet-based cognitive-behavioral therapy (ICBT). It is designed to compare two groups, one group receiving ICBT and attention training towards threat cues and one group receiving ICBT and control training. We hypothesize that the group receiving ICBT plus attention training will show a better treatment outcome on social anxiety measures than the control group.

## Methods

### Design

We will conduct a randomized, controlled, double-blind trial to compare the ICBT-plus-attention-training condition with the ICBT-plus-control-training condition. The study has been approved by the regional ethical board and has been registered in clinicaltrials.gov (NCT01570400). The guidelines for executing and reporting Internet intervention research will be followed [48]. Participants will be requested to give written informed consent.

### Study population

Participants will be adults fulfilling the diagnostic criteria of SAD according to the DSM-IV [49]. Participants meeting diagnostic criteria for other co-morbid disorders will be included, as long as SAD can be considered the primary diagnosis and complaint. Hence, participants with acute substance-use disorder, psychotic symptoms, or bipolar disorder will be excluded. Criteria for inclusion will be the following: (a) being at least 18 years old; (b) having access to the Internet; (c) being able to take part in a telephone-administered diagnostic interview; (d) meeting diagnostic criteria for a primary diagnosis of SAD; (e) not participating in any other psychological treatment for the duration of the study; and (f) if on prescribed medication for anxiety or depression, dosage has to be constant for 3 months prior to the start of the treatment. Participants with suicidal thoughts, defined as a score of four or higher on item 9 of the Montgomery Åsberg Rating Scale-Self-rated (MADRS-S, [50]) will be interviewed by phone using the SAD PERSONS interview [51] to evaluate their suicidal risk. Participants with a suicidal risk will be excluded from the study and will be referred to local psychiatrists or psychologists.

### Sample size

Effect sizes of the difference between ICBT plus attention training and ICBT plus control training are difficult to estimate. First, in previous trials, the pure comparison of attention training versus control training resulted in varied controlled effect sizes between *d* = −0.07 and 1.59 [34,40]. Second, ICBT alone yielded large effect sizes of *d* = 0.70 to 1.38 compared to a waiting-list control (e.g.[11,52]). The combination of these two approaches has not yet been evaluated systematically. By considering clinical relevancy, we based our sample size calculation on a medium controlled effect size. On the basis of a controlled effect size of *d* = 0.50 in a two-sided *t*-test (α = 0.05, power 80%) comparing ICBT plus attention training with ICBT plus control training, the sample size, *N*, will be set at 128 participants, with 64 participants in each group.

### Recruitment and procedure

The selection of the participants will follow two steps. First, participants will be asked to fill in a computerized screening battery consisting of the self-rated version of the Liebowitz social anxiety scale-self-report (LSAS-SR) [53], the Social Interaction Anxiety Scale (SIAS) [54], the Social Phobia Scale (SPS) [54], the Montgomery and Åsberg Depression Rating Scale self-rated version [50], the Quality of Life Inventory [55], and additional questions regarding current and past treatment. Finally, a cognitive bias task, consisting of 96 trials, will be administered, to obtain baseline data on biases. In a second step, participants who score above the cut-off of 30 on the LSAS-SR will be invited to take part in a telephone-administered diagnostic interview. Two advanced MSc clinical psychology students will conduct the Structured Clinical Interview for DSM-IV Axis I Disorders [56]. Both interviewers are trained in using the SCID-I. Participants fulfilling the criteria of SAD as a primary diagnosis and meeting all other inclusion criteria will then be randomized by an online true random-number service independent of the investigators and therapists. Participants, investigators, and Internet therapists will remain blind to the randomized group affiliation throughout the trial.

After the pre-assessment and the randomization procedure, participants will receive access to a website that will present the tasks of either the attention-training program or the control training program, as appropriate, as well as the CBT self-help manual. The combined intervention will take 11 weeks. During Weeks 1 and 2, participants will be asked to carry out the attention training or control training exercises once a day for a total of 14 days. From Week 3 to Week 11, participants will be asked to complete the nine modules of the CBT self-help manual, with one module each week.

Primary and secondary outcome questionnaires will be completed prior to the intervention (Week 0), after the first intervention at the end of Week 2, and at the end of Week 11, regardless of how many exercises and modules have been completed, as well as four months after the intervention. Additionally, the attention bias will be assessed prior to and after the intervention. All assessments will be administered via the Internet, using a procedure with appropriate psychometric properties [57-59]. All communication will be administered via an online messaging system resembling standard emailing systems connected to the study [60]. All data are encrypted in the database and a cryptographic protocol (Secure Sockets Layer) will be used to provide communication security over the Internet.

### Intervention

All participants will receive the same CBT-self-help manual. In addition, participants will receive attention training or control training exercises depending on their group affiliation.

### Attention training and control training

Participants will be randomly assigned to either a 14-day long real attention modification program or a placebo condition. Both conditions will be identical except for the location of the probe. Hence, in both conditions a trial begins with a 500 ms inter-trial pause consisting of a blank white screen (#FFFFFF), followed by a black fixation cross (“+”) presented in the center of the screen for 500 ms (Arial size 14 and black font color). Immediately following the termination of the fixation cue, a web-based Flash program in full screen mode will present a pair of stimuli. These will be either two words (Arial size 16 in black font color) with different emotional valences, or two portrait images of the same person’s face expressing two different facial expressions (200 pixels high; width 131, 133 or 148 pixels, depending on stimulus set).

The pair of stimuli, presented spatially separated, one above the other in the center of the screen, will, for each trial, be randomly chosen from one of three possible combinations: positive-neutral, positive–negative, or neutral-negative. Each combination will be used an equal number of times during a session. The relative spatial order of the two constituent stimuli will also be randomly chosen with equal probability. For the first 96 trials of each session, these pairs will be displayed for 1000 ms. For the 96 remaining trials of treatment and placebo sessions the pairs will be displayed for 500 ms.

After each pair of stimuli has been displayed, it will be replaced with a probe, taking with equal probability, the position of the upper or the lower previously displayed stimulus. The probe will be an arrow (Arial size 16 in black font color), pointing to either the left (<) or the right (>). Participants are instructed to respond as quickly as possible, but without making mistakes, to the direction of the arrow by pressing the corresponding arrow key on the keyboard. The probe will remain on the screen until a response is given, after which the next trial will begin.

The stimuli will consist of images from 62 men and 62 women, each showing three different facial expressions: positive (happiness), neutral, and negative (disgust), as well as of 111 positive words, 111 neutral words, and 111 negative words. Both the faces and the words are centered on the screen with the faces 4 pixels and the words 180 pixels apart. The stimuli material will be taken from the Umeå University Database of Facial Expressions [61], the Karolinska Directed Emotional Faces [62] and the Matsumoto and Ekman’s Japanese and Caucasian Facial Expressions of Emotion [63].

In the treatment condition, the probe will always be at the same location as the more negative stimulus in the pair. In contrast, in the placebo condition the probe appears randomly, with equal frequency in the two positions. The intervention will consist of 14 sessions of either treatment or placebo exercises. As shown in Table 1, each session will encompass 192 trials.

**Table 1:** Number of trials per probe position per session depending on condition including assessment phase

	**Trials per session**	**Length of stimulus display, ms**	**positive - neutral**	**positive - negative**	**neutral - negative**	**positive- neutral**	**positive- negative**	**neutral- negative**
**Bias assessment**	96	500 (faces)	8	8	8	8	8	8
		500 (words)	8	8	8	8	8	8
**Control training**	192	500 (faces)	8	8	8	8	8	8
		500 (words)	8	8	8	8	8	8
		1000 (faces)	8	8	8	8	8	8
		1000 (words)	8	8	8	8	8	8
**Attention training**	192	500 (faces)	16	16	16	-	-	-
		500 (words)	16	16	16	-	-	-
		1000 (faces)	16	16	16	-	-	-
		1000 (words)	16	16	16	-	-	-

Underlined words indicate the position of the probe.

The attention bias will be assessed in two additional sessions, one before (day 0) and one after (day 15) the training. The attention-bias assessment includes 96 dot-probe trials similar to those of the training sessions, with the cue presented for 500 ms. The probe will appear with equal frequency at the location of neutral, negative, and positive cues (see Table 1).

### Cognitive-behavioral self-help

The cognitive-behavioral self-help intervention will consist of our previously evaluated self-help manual for SAD, which consists of 186 pages divided into nine chapters (modules) adapted for use over the Internet [11,13]. The introductory module describes SAD and CBT. Modules 2 to 4 describe a cognitive model for SAD and introduce cognitive restructuring. Modules 5 to 7 introduce exposure exercises and exercises on self-focused attention. Modules 8 and 9 mainly cover social skills and relapse prevention. Each module consists of information and exercises (homework assignments) and ends with a short quiz to check adherence. Participants will be asked to summarize, in their own words, a central section of the module in question and to describe the outcome of the exercises in weekly email correspondence with their Internet therapist. Internet therapists will be six MSc clinical psychology students, trained and supervised by a licensed clinical psychologist. Therapists will give feedback on the homework assignment within 24 h. When the homework is completed, the next module will be made accessible. Alternatively, instruction on what is needed to proceed to the next module will be sent to the participant. Participants will have access to an online discussion forum. For each module, participants will be asked to post at least one message about a topic related to the module and to share their weekly achievements with the rest of the group. They will also be encouraged to provide feedback and support for others. Discussions will be surveyed but the study personnel will not take part in them.

### Outcome measures

We will use a composite score of the following three self-report measures of social anxiety as primary outcome measure: the self-report version of the Liebowitz Social Anxiety Scale [53], the SPS, and the SIAS [54]. In addition, as secondary outcome measures, we will administer the MADRS-S to assess depressive symptoms [50], and the Quality of Life Inventory [55]. To monitor the progress of social anxiety during the attention and control training exercises, we will administer the Mini-SPIN, a three-item screening measure, prior to each attention or control session [64].

### Statistical analysis

All analyses of primary and secondary outcome measures will be conducted as intention-to-treat analyses (ITT). Depending on the amount of drop-out, the ITT analyses will be carried out either as mixed models or using the ‘last observation carried forward’ method. Social anxiety measures will be integrated in a social anxiety composite following the procedure recommended by Rosnow and Rosenthal [65] and applied by Clark *et al.*[66]. The composite score will be generated by converting each social phobia scale across all assessment points to *z*-scores, and then by averaging across the measures. Between-group changes at post-treatment and at 4-month follow-up on the social anxiety composite and on secondary outcome measures will be calculated using analysis of covariance (ANCOVA), with pre-treatment scores as the covariate. Effect sizes between and within the two groups will be calculated with Cohen’s *d* computed with the pooled standard deviation.

Clinically significant change at post- and follow-up assessment will be estimated for the LSAS-SR, the SIAS, and the SPS. In accordance with Jacobson and Truax [67], we will calculate the proportion of improved and recovered participants using the Reliable Change Index and cut-off scores based on the Jacobson and Truax’s formula, *c*.

The attention-bias assessment will produce reaction times for each participant to three kinds of negatively cued trial (positive–negative, neutral-negative, positive-neutral) and to three kinds of positively cued trial (positive–negative, neutral-negative, positive-neutral). We will calculate the mean reaction time for each participant for positively cued and negatively cued trials, eliminating response latencies for inaccurate trials and response latencies less than 200 ms or greater than 2000 ms. To examine changes in attention bias, we will conduct a 2 (pre-assessment/post-assessment) × 2 (attention training/control condition) × 2 (positive/negative) analysis of variance (ANOVA). Furthermore, we will analyze changes in the attention bias from pre- to post-assessment on an individual level using an attention bias score. We will calculate this bias score by subtracting the mean reaction times to negative cues from the mean reaction times to positive cues. Positive individual bias scores will indicate biased attention towards threat. Negative bias scores will indicate biased attention away from threat.

## Discussion

The planned study aims at evaluating the combination of an attention modification training program with an established Internet-based CBT self-help program. Cognitive bias modification programs are theoretically well suited for integration in CBT. Biases in interpretation and attention processes are part of the cognitive model of SAD [21,68]. This model of maintenance is, at the same time, a model of change. All factors of the model are potential starting points of change and most of them are explicitly addressed in CBT. However, so far, the attentional avoidance of external threat cues has not been integrated in CBT manuals. This is certainly due to the implicit nature of this particular maintaining factor. Individuals are hard put to modify their allocation of attention consciously. At this point, the computerized attention training applied in this study offers a very straightforward opportunity to account for the attention bias.

On the other hand, trials on computerized attention training have not yet aimed at integrating them into standard CBT. This may be because bias modification programs were primarily developed to prove the causal relationship of cognitive biases and psychological symptoms [69]. They were not at first designed to treat patients suffering from these symptoms. Only recently has interest in their therapeutic potential arisen and first studies evaluated their efficacy as stand-alone intervention. The findings of this research design will provide an important further step towards an understanding of the potential of CBM. The design also offers a pathway to address, for the first time, an integral part of the cognitive model, not yet covered by CBT manuals, in its interaction with other maintaining factors, such as self-focused attention and safety behaviors.

Our study aims to compare two groups. In addition to the CBT self-help program, one group will receive attention-training exercises, while the other group will receive control training exercises. This randomized add-on design will allow us to draw conclusions about the specific efficacy of ICBT plus attention training compared with ICBT plus control training. It will not, however, allow us to estimate the efficacy of the addition of *any* training compared to *no* training. This comparison would require the inclusion of an additional group receiving ICBT alone. As this would lead to a substantial increase in the required sample size and the required therapist time, we had to decide against it. This is certainly a limitation of our study, which can only partly be addressed by the comparison of the achieved effect sizes with those of previous trials on the ICBT program.

Another limitation of the current design is that the efficacy of the applied attention training as stand-alone intervention has only been demonstrated in one prior study (Carlbring *et al*., in preparation). Prior research has focused on attention-training programs that facilitate disengagement from threat. However, as the training program suggested by Amir *et al*. [34] has not proven efficacious in the Internet-based setting in two randomized controlled trials, we decided to opt for the training protocol that actually produced positive results for participants in a previous Internet-based trial. Based on these tentative empirical data and the strong theoretical basis, we hypothesize that the integration of attention training into the CBT rationale will have a beneficial effect. If the combination of ICBT and attention training will prove successful in the planned study and following replications, the implication would clearly be to aim at integrating attention-bias exercises into ICBT as well as into face-to-face therapies. The standardized and computerized nature of the attention-bias exercises would allow the realization of this implication with little difficulty.

### Trial status

Participant recruitment began on 1 August 2012.

## Abbreviations

ANCOVA: Analysis of covariance; ANOVA: Analysis of variance; CBM: Cognitive bias modification; CBT: Cognitive-behavior therapy; DSM-IV: *Diagnostic and Statistical Manual of Mental Disorders*, 4th edition; ICBT: Internet-biased cognitive-behavioral therapy; ITT: Intention-to-treat; JACFEE: Japanese and Caucasian Facial Expressions of Emotion; LSAS-SR: Liebowtiz Social Anxiety Scale-Self-Report; MADRS-S: Montgomery Åsberg Rating Scale-Self-rated; SAD: Social anxiety disorder; SIAS: Social Interaction Anxiety Scale; SPS: Social Phobia Scale.

## Competing interests

The authors declare that they have no competing interests.

## Authors’ contributions

JB, GA, and PC conceived the trial. JB and PC designed the trial. JB and PC drafted this manuscript. GA provided critical review of this manuscript. All authors read and approved the final manuscript.
